# Metaplastic breast carcinoma with osteosarcomatous differentiation: a case report

**DOI:** 10.3389/fonc.2026.1883268

**Published:** 2026-06-30

**Authors:** Xiang Zhao, XiaoYu Sun, Jiaqi Liu

**Affiliations:** 1 School of Nursing, Qilu Medical University, Zibo, China; 2 Department of Pathology, Zibo Central Hospital, Zibo, China; 3 Department of Breast and Thyroid Surgery, Zibo Central Hospital, Zibo, China

**Keywords:** local recurrence, metaplastic breast carcinoma, osteosarcoma, osteosarcomatous differentiation, SATB2, Triple-negative breast cancer

## Abstract

**Background:**

Metaplastic breast carcinoma (MBC) is a rare and aggressive malignancy accounting for less than 0.2-1% of all breast cancers. MBC with osteosarcomatous differentiation is extremely rare (0.003%-0.12%) and presents significant diagnostic and therapeutic challenges.

**Case presentation:**

We report a case of a 65-year-old female with history of breast-conserving surgery for left breast invasive carcinoma in 2021, who presented with a recurrent left breast mass in December 2025. After adjuvant therapy including ACT chemotherapy, radiotherapy and capecitabine, imaging revealed a 4.47 x 3.4 x 2.8 cm irregular mass with calcifications. Core needle biopsy suggested metaplastic carcinoma. After 4 cycles of TP plus immunotherapy showing stable disease, modified radical mastectomy was performed. Final pathology confirmed MBC with osteosarcomatous differentiation (4.5 x 3 x 3 cm), triple-negative with SATB2 positivity and Ki-67 70%. Postoperative BRCA1/2 testing was negative, and adjuvant AC chemotherapy combined with immunotherapy was administered.

**Conclusion:**

This case highlights the diagnostic challenges and treatment considerations for MBC with osteosarcomatous differentiation. Multimodality treatment including surgery, anthracycline-based chemotherapy, immunotherapy, and radiotherapy may provide optimal outcomes.

## Introduction

Breast cancer has the highest incidence of all cancers affecting women worldwide (1). Metaplastic breast carcinoma (MBC) is a heterogeneous group of indolent to highly aggressive tumors that consists of epithelial and non-glandular elements such as matrix-production, spindle cell morphology, squamous differentiation, or heterologous differentiation (i.e., osseous or chondroid) (2). Of the breast carcinoma subtypes, MBC accounts for less than 0.2-1% of all cases of invasive breast cancer (3, 4).

In contrast to low-grade variants like fibromatosis-like metaplastic carcinoma, a subset of these rare tumors is highly aggressive with poor prognosis and high mortality rate (5, 6). MBC is usually triple negative and in general portends a worse prognosis and 5-year survival than triple-negative breast carcinomas without metaplastic features (4, 7). Osseous and sarcomatous differentiation are the rarest mesenchymal subtypes of MBC and are typically poorly differentiated (8) with osteosarcomatous differentiation accounting for between 0.003% and 0.12% of all breast cancer cases (9, 10). We present a case of a 65-year-old female with a history of previously treated left invasive breast carcinoma with subsequent diagnosis of a triple negative metaplastic breast carcinoma with osteosarcomatous differentiation approximately 4 years after initial breast cancer diagnosis.

## Case report

### Patient history

Our patient was a 65-year-old female with a history of left breast carcinoma initially diagnosed and treated at Zibo First Hospital in October 2021. According to the previous medical records, the initial tumor was diagnosed as left breast invasive carcinoma with a triple-negative phenotype, measuring 2.5 × 2.0 × 2.0 cm. She underwent breast-conserving surgery and sentinel lymph node biopsy, with no sentinel lymph node metastasis identified (0/4). Postoperatively, she received eight cycles of adjuvant ACT chemotherapy, including pegylated liposomal doxorubicin, cyclophosphamide, and nab-paclitaxel, followed by adjuvant radiotherapy to the left breast (25 fractions, total dose 50 Gy) and low-dose capecitabine (650 mg/m², twice daily) for one year.

In December 2025, the patient presented to Zibo Central Hospital for evaluation of a newly palpable mass in the left breast. Further imaging and pathological assessment were subsequently performed at our institution. Because the initial surgery was performed at another hospital, the original histological slides and paraffin blocks from the 2021 tumor were not available for review at our institution.

### Diagnostic assessment

In December 2025, breast ultrasound demonstrated a hypoechoic nodule with unclear boundaries, irregular shape, and heterogeneous echogenicity in the left breast at 1–3 oclock position near the nipple, measuring approximately 41 x 26 mm (**Figure 1**). Multiple coarse and fine calcifications were observed within the mass. Color Doppler imaging showed abundant blood flow signals. No significant lymphadenopathy was noted in both axillae.

**Figure 1:**
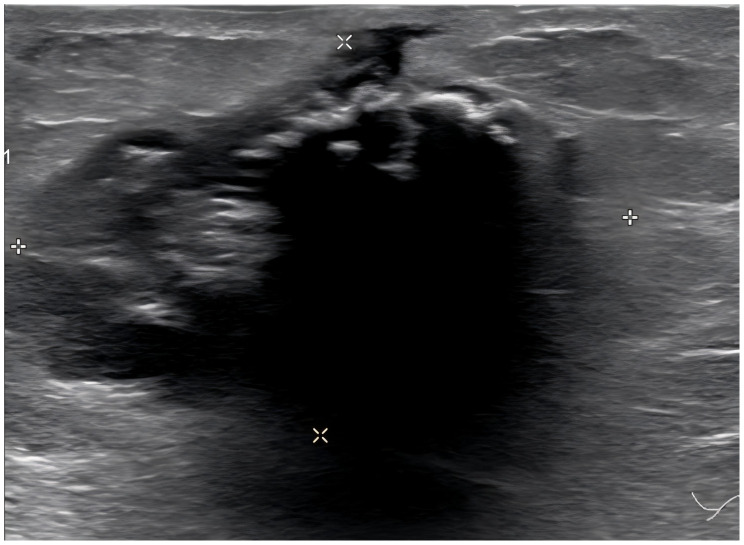
Breast ultrasonography showing a hypoechoic nodule (41 × 26 mm) with unclear boundaries, irregular shape, heterogeneous echogenicity, and multiple calcifications in the left breast.

Breast MRI demonstrated an irregular mass (2.6 x 2.2 cm) in the deep portion of the left breast outer quadrant. The mass showed mixed signal intensity on T2-weighted imaging (heterogeneous iso- to hyperintense), low signal on T1-weighted imaging, high signal on DWI, and slightly low signal on ADC map (ADC value approximately 1.36). The lesion exhibited skin thickening and retraction, involving the adjacent chest wall (**Figure 2**). Dynamic contrast-enhanced MRI showed marked heterogeneous enhancement with time-signal intensity curves showing plateau and washout patterns.

**Figure 2:**
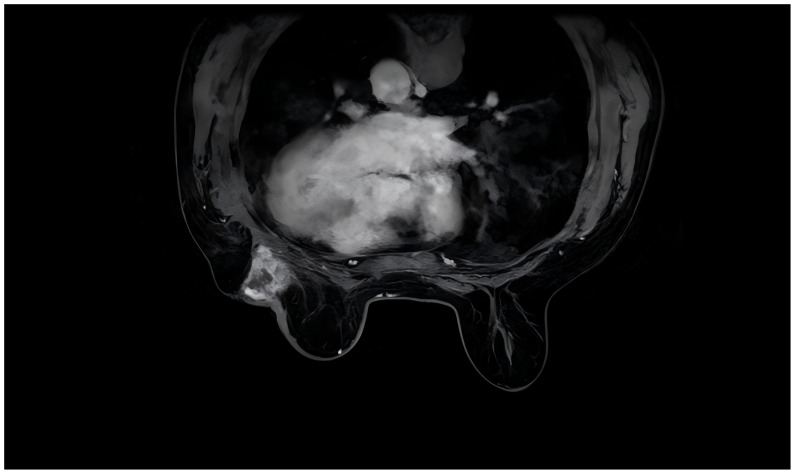
Breast MRI demonstrating an irregular mass (2.6 × 2.2 cm) with skin thickening and chest wall involvement.

Ultrasound-guided core needle biopsy demonstrated atypical glands with stromal spindle cell proliferation and focal calcification. Immunohistochemistry suggested metaplastic carcinoma: TRPS-1 (+), Vimentin (partially +), CKAE1/AE3 (partially +), CK5/6 (-), P63 (focally +), SMA (-), CD34 (-), ER (-), PR (-), HER-2 (2+ in epithelial cells, 0 in spindle cells), P53 (+++, 90%), Ki-67 (+, 55%).Subsequent fluorescence *in situ* hybridization (FISH) testing confirmed the absence of HER2 gene amplification. Supplemental immunohistochemistry showed PD-L1 (E1L3N) with TPS 1% and IC <1%.CPS (Comprehensive Positive Score): 1.

### Therapeutic intervention

The patient received treatment with TP regimen plus immunotherapy (nab-paclitaxel + carboplatin + toripalimab) for 4 cycles for recurrent disease. After 4 cycles of treatment, imaging evaluation showed stable disease (SD) with minimal change in tumor size. According to the 2025 CSCO Breast Cancer Guidelines, after multidisciplinary team (MDT) consultation, the patient underwent modified radical mastectomy in March 2026.

Postoperative BRCA1/2 gene testing was negative. Based on postoperative pathology results and further MDT consultation with extensive literature review, the decision was made to continue adjuvant chemotherapy with AC regimen for 4 cycles combined with immunotherapy and radiotherapy. This treatment approach was supported by evidence showing that osteosarcoma components are sensitive to anthracycline-based chemotherapy (21, 22).

### Follow-up and outcomes

The surgical specimen revealed a malignant breast tumor (4.5 x 3 x 3 cm) diagnosed as metaplastic carcinoma with predominant mesenchymal differentiation (heterologous osteosarcoma)(**Figure 3**). The tumor showed partial degeneration, cystic changes, and scattered minimal lymphocyte infiltration in the stroma, consistent with Miller-Payne Grade 2 response. All margins were negative. All 12 axillary lymph nodes were negative for metastasis.

**Figure 3:**
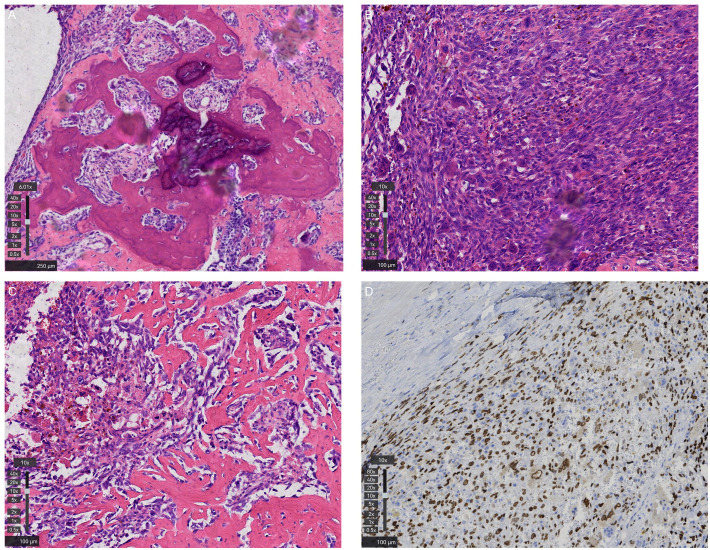
Histological features and SATB2 expression in metaplastic carcinoma of the breast. **(A)** H&E staining at 60× magnification (scale bar = 250 μm) showing osteoid matrix in metaplastic carcinoma. **(B)** H&E staining at 100× magnification (scale bar = 100 μm) displaying a sarcomatoid component. **(C)** H&E staining at 100× magnification (scale bar = 100 μm) illustrating the transitional zone between the sarcomatoid area and fibro-osteoid matrix. **(D)** Immunohistochemical SATB2 staining at 100× magnification (scale bar = 100 μm) demonstrating nuclear positivity in the sarcomatoid tumor cells.

Immunohistochemistry showed: CKAE1/AE3 (partially +), EMA (partially +), Vimentin (+), GATA3 (partially +), Trps-1 (+), CK5/6 (focally +), P63 (partially +), ER (-), PR (-), HER-2 (0), CD34 (-), SATB2 (+)(**Figure 3**), S-100 (partially +), SMA (-), desmin (-), beta-Catenin (membranous +), P53 (+++, 90%), Ki-67 (+, 70%)(**Figure 4**). The SATB2 positivity confirmed osteosarcomatous differentiation (**Figure 3**) (17). The patient tolerated the treatment well without major complications.Treatment timeline of the patient is showing in picture (**Figure 5**).

**Figure 4:**
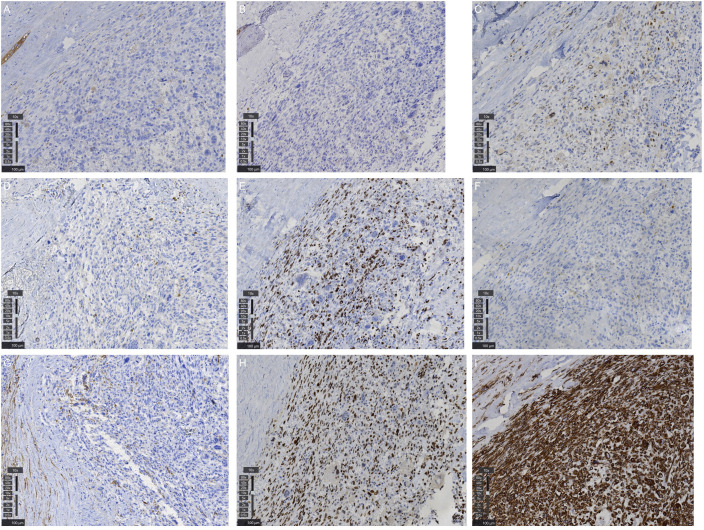
Immunohistochemical profile of metaplastic carcinoma of the breast. **(A)** CKpan IHC, 100× (scale bar = 100 μm), negative in tumor cells with internal positive control. **(B)** ER IHC, 100× (scale bar = 100 μm), negative. **(C)** GATA3 IHC, 100× (scale bar = 100 μm), nuclear positivity. **(D)** HER2 IHC, 100× (scale bar = 100 μm), negative. **(E)** Ki67 IHC, 100× (scale bar = 100 μm), nuclear positivity. **(F)** PR IHC, 100× (scale bar = 100 μm), negative. **(G)** SMA IHC, 100× (scale bar = 100 μm), negative. **(H)** TRPS1 IHC, 100× (scale bar = 100 μm), nuclear positivity. **(I)** Vimentin IHC, 100× (scale bar = 100 μm), membranous positivity.

**Figure 5:**
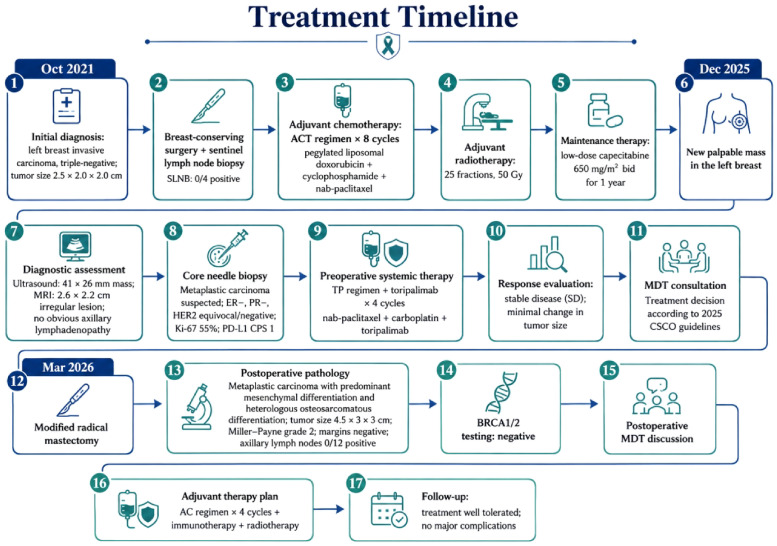
Treatment timeline of the patient.

### Summary of previously reported single-case reports

Compared with previously reported single cases, the present case shares several clinicopathological features, including a breast mass with calcifications, epithelial and osteosarcomatous components, SATB2 positivity, and aggressive biological behavior (**Table 1**). However, our patient was older and had a history of previously treated ipsilateral triple-negative breast carcinoma. Unlike the rapidly progressive case reported by Do et al., our patient had no axillary lymph node metastasis, achieved complete surgical resection with negative margins, and remained under close follow-up after postoperative multimodality treatment. Nevertheless, the triple-negative phenotype, high Ki-67 index, local recurrence, and limited response to preoperative TP-based chemoimmunotherapy suggest a high risk of recurrence and support the need for close long-term surveillance.

**Table 1:** Reported single cases of metaplastic breast carcinoma with osteosarcomatous/osseous differentiation with available treatment and follow-up data.

Author, year	Age, sex	Tumor size	Receptor status	Key pathological features	Treatment received	Follow-up/outcome
Yang et al., 2022 (10)	42, female	2.7 x 2.2 x 1.7 cm	IDC component: ER/PR positive; osteosarcomatous component: ER/PR/HER2 negative; SATB2 positive	Mixed metaplastic carcinoma composed of invasive ductal carcinoma and osteosarcoma; osteosarcomatous component accounted for 65%	Radical operation followed by 8 cycles of chemotherapy	Alive without recurrence at 20 months
Do et al., 2024 (26)	59, female	Initially 4.5 x 5.3 cm; progressed to a giant tumor approximately 20 cm	ER-, PR-, HER2-; vimentin positive; CKA positive	Advanced metaplastic breast carcinoma with extensive osseous differentiation; synchronous contralateral breast cancer	Neoadjuvant docetaxel plus cyclophosphamide; toilet mastectomy; carboplatin x3; gemcitabine x3; palliative care	Lung metastasis; progressive disease; died shortly after palliative care
Present case	65, female	4.5 x 3 x 3 cm	ER-, PR-, HER2-; SATB2 positive; Ki-67 70%	Recurrent metaplastic breast carcinoma with predominant heterologous osteosarcomatous differentiation	TP plus toripalimab; modified radical mastectomy; postoperative AC plus immunotherapy and radiotherapy	No major complications; under close follow-up

Primary osteosarcoma of the breast and malignant phyllodes tumor with osteosarcoma component were not included because they represent differential diagnoses rather than the same disease entity.

## Discussion

### Clinical and imaging presentation

Metaplastic breast carcinoma often clinically presents as a large palpable mass ranging from 1 to 21 cm (2), which was observed in this case. The maximum dimension of the mass was 4.5 cm by surgical pathology. Other reports of MBC describe mass measurements at initial presentation as greater than 2 cm (9–15).

In our case, the tumor presented as a hypoechoic nodule with irregular shape, unclear boundaries, and multiple calcifications on ultrasound, which is consistent with previous reports of MBC with osteosarcomatous differentiation (9, 10, 16). The presence of calcifications on imaging is a characteristic finding in MBC with osseous differentiation.

### Pathological features

The pathological hallmark of MBC with osteosarcomatous differentiation is the presence of both epithelial and mesenchymal components. In our case, the tumor showed two distinct components: areas of invasive carcinoma and areas of osteosarcoma with SATB2 positivity. SATB2 (Special AT-rich sequence-binding protein 2) is a nuclear marker specifically expressed in osteoblasts and is considered a reliable marker for osteoblastic differentiation (17, 18). SATB2 positivity in our case confirmed the osteosarcomatous component.

MBC with osteosarcomatous differentiation is typically triple-negative (ER-, PR-, HER-2-), which was observed in our case (4, 7). The high Ki-67 proliferation index (70%) indicates aggressive tumor biology and poor prognosis.

### Differential diagnosis

The main differential diagnoses include:

1. Primary breast osteosarcoma: A pure mesenchymal malignancy without epithelial components. The presence of epithelial components confirmed by CKAE1/AE3 and EMA positivity in our case supports the diagnosis of MBC rather than primary osteosarcoma (10, 19).
2. Malignant phyllodes tumor with osteosarcoma component: CD34 positivity typically favors phyllodes tumor, while CD34 negativity in our case argued against this diagnosis (20).
3. Metaplastic carcinoma: The presence of both epithelial and mesenchymal components confirms the diagnosis of MBC.

### Treatment

The optimal treatment for MBC with osteosarcomatous differentiation remains challenging due to its rarity. Surgical resection with clear margins is the primary treatment approach (21, 22). The responsiveness of MBC with osteosarcomatous or osseous differentiation to chemotherapy and radiotherapy remains uncertain. Several reports have suggested limited sensitivity to conventional chemotherapy, particularly in the neoadjuvant setting (5, 6, 26). Do et al. reported disease progression despite neoadjuvant docetaxel plus cyclophosphamide and subsequent palliative carboplatin and gemcitabine, and concluded that the role of neoadjuvant chemotherapy in this histopathological subtype may be limited and should be carefully considered (26). However, other cases have shown more favorable short-term outcomes after surgery and adjuvant chemotherapy, such as the case reported by Yang et al., in which the patient remained recurrence-free at 20 months after surgery and eight cycles of chemotherapy (10). Therefore, current evidence does not support a uniform conclusion regarding chemotherapy sensitivity, and treatment decisions should be individualized according to tumor stage, pathological composition, receptor status, prior treatment response, resectability, and multidisciplinary team assessment (10, 25, 26).

In our case, after 4 cycles of TP plus immunotherapy, the tumor showed minimal response (stable disease), which is consistent with the known chemoresistance of MBC (5, 6). Following MDT discussion, modified radical mastectomy was performed. Based on postoperative pathology and literature review showing that osteosarcoma components are sensitive to anthracycline-based chemotherapy (21–24), we selected AC regimen as adjuvant chemotherapy combined with immunotherapy.It should be acknowledged that direct evidence supporting anthracycline-based chemotherapy specifically for metaplastic breast carcinoma with osteosarcomatous differentiation remains limited due to the extreme rarity of this entity. In the present case, the treatment decision was made after multidisciplinary team discussion and was based on individualized biological and clinical considerations. First, the SATB2-positive osteosarcomatous component showed morphological and immunohistochemical features of osteoblastic differentiation, which provided a biological rationale for considering an anthracycline-based regimen, although this rationale was extrapolated from evidence in conventional osteosarcoma rather than directly established in this rare breast cancer subtype. Second, the tumor showed aggressive clinicopathological features, including triple-negative status, a high Ki-67 index of 70%, local recurrence, and limited response to prior TP-based chemoimmunotherapy, supporting the need for further systemic treatment. Third, the final therapeutic strategy incorporated available literature, the patient’s prior treatment history, postoperative pathological findings, and MDT judgment. Therefore, although the use of AC chemotherapy in this case was biologically justifiable, it should be interpreted as an individualized treatment decision rather than an established standard of care. Prospective evidence specifically evaluating anthracycline-based chemotherapy in MBC with osteosarcomatous differentiation is lacking, and current evidence remains mainly derived from case reports, small retrospective series, and extrapolation from osteosarcoma treatment principles.

### Prognosis

MBC is associated with a poorer prognosis compared to conventional invasive breast carcinoma of no special type of similar stage (4, 7). MBC with osteosarcomatous or osseous differentiation represents an exceptionally rare subtype, and the available evidence is mainly derived from isolated case reports, small retrospective series, and literature reviews (9, 10, 12, 25–27). Previous reports suggest that this subtype often presents as a rapidly growing breast mass, frequently with calcifications, and is commonly associated with aggressive biological behavior (9, 10, 12, 26, 27). However, reported outcomes are variable, reflecting the rarity and histological heterogeneity of the disease, differences in tumor stage and size, the proportion of epithelial and osteosarcomatous components, and the lack of standardized systemic treatment (2, 7, 8, 25, 26).

The pattern of disease spread may also differ from that of conventional breast carcinoma. Although MBC can present with large tumors, axillary lymph node involvement appears less frequent than in conventional invasive breast cancer, whereas hematogenous metastasis, particularly to the lung and, less frequently, bone, has been repeatedly described (5, 6, 9, 26). Do et al. reviewed reported cases of MBC with osseous differentiation and noted that most patients did not have lymph node metastasis, surgery was the main treatment modality, and the lung was the most common metastatic site (26). In their own case, despite neoadjuvant docetaxel plus cyclophosphamide, surgery, carboplatin, and gemcitabine, the tumor progressed with lung metastasis and the patient died shortly after palliative care, highlighting the potentially aggressive and chemoresistant nature of this subtype (26).

Nevertheless, the prognosis is not uniformly poor in all reported cases. Yang et al. reported a 42-year-old patient with mixed metaplastic carcinoma composed of invasive ductal carcinoma and a predominant osteosarcomatous component. The invasive ductal carcinoma component was ER/PR positive, whereas the osteosarcomatous component was ER/PR/HER2 negative and SATB2 positive. After surgery and completion of eight cycles of chemotherapy, the patient remained alive without recurrence during 20 months of follow-up (10). This comparatively favorable outcome suggests that clinical behavior may vary substantially among individual cases and may be influenced by tumor burden, completeness of surgical resection, metastatic status, pathological composition, and postoperative treatment (10, 25, 26).

In the present case, several features suggested a high risk of recurrence, including triple-negative phenotype, high Ki-67 proliferation index of 70%, local recurrence after prior breast-conserving surgery and radiotherapy, and limited response to preoperative TP-based chemoimmunotherapy. However, complete surgical resection with negative margins and the absence of axillary lymph node metastasis were favorable pathological factors. Given the rarity of MBC with osteosarcomatous differentiation and the variability of outcomes reported in the literature, long-term surveillance is essential for early detection of local recurrence and distant metastasis (9, 10, 25–27).

## Conclusion

This case report highlights the importance of understanding the clinical presentation and imaging features of metaplastic breast carcinoma given its aggressive nature and treatment implications. It also adds to the available data on metaplastic breast carcinoma with osteosarcomatous differentiation in the initial presentation, imaging features, and features of disease progression.

Due to its rarity and aggressive behavior with high risk of distant metastasis, accurate diagnosis and appropriate management are crucial. Multimodality treatment including surgery, chemotherapy, immunotherapy, and radiotherapy may provide optimal outcomes. The role of anthracycline-based chemotherapy should be further investigated for MBC with osteosarcomatous differentiation.

## Data Availability

The raw data supporting the conclusions of this article will be made available by the authors, without undue reservation.
